# Effects of Continuous LPS Induction on Oxidative Stress and Liver Injury in Weaned Piglets

**DOI:** 10.3390/vetsci10010022

**Published:** 2022-12-29

**Authors:** Yunxiao Zhou, Xiaofen Hu, Shengwei Zhong, Wanting Yu, Jue Wang, Wenlu Zhu, Tingyu Yang, Guotong Zhao, Yijie Jiang, Yong Li

**Affiliations:** College of Animal Science and Technology, Jiangxi Agricultural University, Nanchang 330045, China

**Keywords:** lipopolysaccharide, piglet, oxidative stress, liver injury, endotoxin tolerance

## Abstract

**Simple Summary:**

Pigs have been evaluated as good model animals for human physiological and pathological research. During the weaned period, piglets are vulnerable to pathogens due to the transformation from a liquid to a solid feed, environmental changes, and an immature autoimmune system. Intraperitoneal injection of lipopolysaccharide (LPS) has been widely applied to simulate liver injury in piglets, but there are still some puzzles and gaps in our understanding. Therefore, we explored the effects of continuous low-dose LPS induction on serum enzyme activity, antioxidant level, liver morphology, and mRNA expression related to liver injury in weaned piglets. The findings demonstrated that oxidative damage and liver injury occurred at the early stage with LPS induction, but the damage was gradually weakened at the later stage. This indicated that continuous LPS induction may cause endotoxin tolerance after a certain amount of time.

**Abstract:**

Due to imperfections in their immune and digestive systems, weaned piglets are susceptible to invasions of the external environment and diseases, especially bacterial infections, which lead to slow growth, tissue damage, and even the death of piglets. Here, a model of weaned piglets induced by *Escherichia coli* lipopolysaccharide (LPS) was established to explore the effects of continuous low-dose LPS induction on the mechanism of liver injury. A total of forty-eight healthy 28-day-old weaned piglets (weight = 6.65 ± 1.19 kg) were randomly divided into two groups: the CON group and LPS group. During the experimental period of thirteen days, the LPS group was injected intraperitoneally with LPS (100 μg/kg) once per day, and the CON group was treated with the same volume of 0.9% NaCl solution. On the 1st, 5th, 9th, and 13th days, the serum and liver of the piglets were collected for the determination of serum biochemical indexes, an antioxidant capacity evaluation, and histopathological examinations. In addition, the mRNA expression levels of the TLR4 pathway and inflammatory cytokines were detected. The results showed that the activities of aspartate aminotransferase (AST), alanine aminotransferase (ALT), and alkaline phosphatase (ALP) in the serum increased after LPS induction. The activities of total antioxidant capacity (T-AOC) and glutathione peroxidase (GSH-Px) in the serum and liver homogenate of the LPS group were lower than those of the CON group, while the malondialdehyde (MDA) content in the serum and the activities of catalase (CAT) and superoxide dismutase (SOD) in the liver of the LPS group were higher than those in the CON group. At the same time, morphological impairment of the livers occurred, including hepatocyte caryolysis, hepatocyte vacuolization, karyopycnosis, and inflammatory cell infiltration, and the mRNA expression levels of TLR4, MyD88, NF-κB, TNF-α, IL-6, and IL-10 were upregulated in the livers after LPS induction. The above results were more obvious on the 1st and 5th days of LPS induction, while the trend during the later period was not significant. It was concluded that the oxidative stress and liver injury occurred at the early stage of LPS induction, while the liver damage weakened at the later stage. The weaned piglets probably gradually developed tolerance to the endotoxin after the continuous low-dose induction of LPS.

## 1. Introduction

The liver, as the primary metabolic and detoxification organ, is responsible for the excretion of various toxins, blood detoxification, and removal of unwanted drug metabolites accumulated by other tissues [1,2]. Due to its unique anatomical location, the liver is frequently exposed to circulating antigens, endotoxins, and even microorganisms [3]. However, hepatocytes, Kupffer cells, and endothelial sinusoidal cells in the liver are involved in immunity, anti-infection, and metabolic responses through cross-talk and interaction of multiple cells, and they maintain the balance between liver immune tolerance and response, which is essential for host defense and tissue repair in severe infections and sepsis [4]. In animal husbandry, many factors (such as bacterial infection, stress, and feed toxin residue) can lead to liver structural damage, dysfunction, and hepatocyte death, and gut-derived bacterial endotoxin is one of the crucial factors causing various liver diseases [3,5,6]. When the intestinal function is destroyed, it will lead to increased intestinal permeability and translocation of metabolites to the liver, causing liver function damage [7].

Lipopolysaccharide (LPS) is a major constituent of the outer membrane of Gram-negative bacteria, and it is composed of an endotoxic fat-like part and a core sugar constituent consisting of approximately 10 monosaccharides [8]. Under normal conditions, resident macrophages in the liver can effectively remove non-pathogenic amounts of circulating LPS from circulation [9,10,11]. However, when the LPS level is gradually increased beyond the tolerance range of the liver, this will lead to hepatocyte injury and will stimulate liver macrophages to release many inflammatory cytokines and cytotoxic mediators, such as reactive oxygen species (ROSs), which can induce the liver’s immune response and promote liver inflammation, oxidative damage, and cell apoptosis [12,13]. ROSs act as key signaling molecules in the progression of inflammatory diseases, and they include superoxide anions, hydrogen peroxide, and hydroxyl radicals [14]. The excessive production of ROSs can damage the cell membrane through peroxidation, the formation of malondialdehyde (MDA), and the opening of tight junctions between enterocytes, thus causing an increase in intestinal permeability to endotoxin, local or systemic inflammatory reactions, and an imbalance between oxidants and antioxidants in the body [15,16,17].

In addition, the interaction between inflammatory mechanisms and immunomodulators in the liver is essential for maintaining its homeostasis and protecting the body from pathogens and tissue damage [18]. When external stimuli elicit an inflammatory response in the body, immune cells can initiate a signaling cascade that activates key transcription factors, including nuclear factor-κB (NF-κB), mitogen-activated protein kinases, and activator protein 1, which, in turn, regulate inflammation-specific genes [19]. Toll-like receptors (TLRs) play a central role in the activation of the innate system by recognizing the molecular patterns related to bacterial pathogens, in which TLR4, as the immune receptor of LPS, is the main participant, and myeloid differentiation factor 88 (MyD88) is the key adapter molecule of TLR4 signaling [20,21]. LPS can stimulate the over-activation of immune cells, such as macrophages and neutrophils, by recognizing TLR4. Some researchers [22,23] found that when agonistic LPS interacted with the host myeloid differentiation protein 2 (MD-2)/TLR4 complex, it triggered the inflammatory signal cascade through the MyD88-dependent and/or MyD88-independent pathways, which led to the nuclear translocation of transcription factor NF-κB, and then stimulated the synthesis and secretion of proinflammatory cytokines, including tumor necrosis factor-alpha (TNF-α), interleukin-6 (IL-6), and other proinflammatory mediators, so that inflammatory cells could penetrate into target organs, causing dyslipidemia, insulin resistance, and tissue damage. Therefore, LPS can cause excessive and uncontrolled production of inflammatory mediators and can further lead to potentially fatal systemic diseases, such as diabetes, cardiovascular disorders, and septic shock [24,25].

Piglets are more prone to diarrhea, low feed intake, weight loss, and even death due to separation from sows and sudden changes in diet. Meanwhile, piglets’ immune and digestive systems are not mature [26]. A single intraperitoneal injection of LPS has been widely used to imitate a bacterial infection in weaned piglets, but there have been few studies on the damage to animals with continuous LPS injection [27,28]. Here, a model of weaned piglets was established with *Escherichia coli* LPS as a virulence factor. Its purpose was to investigate the effects of continuous low-dose LPS induction on oxidative stress and liver injury in weaned piglets and to comprehend the theoretical mechanism of intestinal bacterial infection for liver injury.

## 2. Materials and Methods

### 2.1. Animals and Experimental Design

Forty-eight healthy 28-day-old weaned piglets (weight = 6.65 ± 1.19 kg, Duroc × Landrace × large white) were purchased from Jiangxi Aoyun Agricultural Development Co. Ltd. (Nanchang, Jiangxi, China), and they underwent a feed supply transition period. After three days of pre-feeding to alleviate any stress responses, piglets were randomly divided into two treatment groups. Among them, the LPS group was injected intraperitoneally with LPS (*Escherichia coli* serotype 055: B5; Cat. No. L2880; Sigma Chemical Inc., St. Louis, MO, USA) at 100 μg/kg body weight every day [29,30]; the control group (CON group) was injected with 0.9% sterile saline at the same volume as that used for the LPS group. The entire experimental period lasted for 13 days, and the piglets were allowed ad libitum access to water and feed. On the 1st, 5th, 9th, and 13th days, six piglets were randomly selected from each group. Three hours after intraperitoneal injection of LPS or saline, blood samples were collected from the anterior vena cava and then centrifuged (3000× *g* rpm, 10 min, 4 °C) to separate the serum, which was stored at −80 °C in a refrigerator (Thermo Fisher Scientific, Waltham, MA, USA) until the analyses of the serum biochemical parameters. After six hours of the LPS challenge, the piglets were killed humanely, and the appropriate livers were immediately taken. A portion of the liver tissue was fixed in 4% paraformaldehyde for at least 24 h before being used for paraffin embedding; the remaining portion was quickly placed in liquid nitrogen and then stored in a refrigerator at −80 °C for further analysis.

### 2.2. Serum Biochemical Index Evaluation

The serum was taken out of the −80 °C ultra-low temperature refrigerator and placed at 4 °C until it melted for biochemical index detection. The activities of serum aspartate aminotransferase (AST, Cat. No. C010-2-1), alanine aminotransferase (ALT, Cat. No.C009-2-1), and alkaline phosphatase (ALP, Cat. No. A059-2) were measured by using a microplate method according to the kit’s instructions (Nanjing Jiancheng Bioengineering Institute Inc., Nanjing, Jiangsu, China). Finally, the absorbances of AST, ALT, and ALP were measured at 510, 510, and 520 nm by using a microplate meter (Molecular Devices Co., Ltd., Shanghai, China), and the serum enzyme activities were calculated by drawing standard curves.

### 2.3. Antioxidant Evaluation

The serum was diluted with saline to a suitable concentration and directly used for the detection of antioxidant indexes. Meanwhile, 10% tissue homogenate was prepared; the liver tissues of the two groups (200 mg) were separately homogenized in a ninefold volume of frozen saline and then centrifuged at 2500× *g* rpm for 10 min at 4 °C to obtain a supernatant for the determination of the liver homogenate concentration and antioxidant index. Protein concentrations in liver homogenate were determined by using the BCA protein concentration detection kit (Wuhan Servicebio Technology Co., LTD., Hubei, China) according to the manufacturer’s instructions. Antioxidant enzyme kits, including total antioxidant capacity (Cat. No. A015-1, T-AOC), superoxide dismutase (Cat. No. A001-3, SOD), catalase (Cat. No. A007-1-1, CAT), glutathione peroxidase (Cat. No. A005, GSH-Px), and malondialdehyde content kits (Cat. No. A003-1, MDA), were purchased from Nanjing Jiancheng Bioengineering Institute Co., Ltd. (Nanjing, Jiangsu, China), and they were used strictly in accordance with the kits’ instructions.

### 2.4. Histopathological Evaluation

Livers were fixed in 4% buffered paraformaldehyde solution for 48–72 h, rinsed with tap water for 12 h, and then dehydrated in increasing concentrations of ethanol (70–100%) solutions with the LEICA ASP200S automatic dehydrator (LEICA Camera AG, Wetzlar, Germany). Subsequently, the tissues were transparentized in xylene and embedded in paraffin; 5 µm sections were sequentially treated with xylene and gradient ethanol and stained with hematoxylin–eosin (H&E) for morphological analysis [31]. Finally, the pathological changes in the liver tissues were evaluated under a light microscope and assessed by using the CellSens Dimension software (Olympus, Tokyo, Japan) for section photography.

### 2.5. Total RNA Isolation and Real-Time Quantitative PCR

The total RNA of the livers was extracted with the Trizol method, and the RNA concentration and optical density values were detected by using an ultraviolet spectrophotometer (Beckman Coulter Inc., Brea, CA, USA). First-strand cDNA was synthesized by using the SweScript All-in-One First-Strand cDNA Synthesis SuperMix Kit (One-Step gDNA Remover) (Wuhan Servicebio Technology Co., LTD., Hubei, China). The first-strand cDNA mix was prepared by combining 4 µL of 5×SweScript All-in-One SuperMix Kit of qPCR, 1 µg of total RNA, and 1 µL of gDNA Remover, and then nuclease-free water was added for a final volume of 20 µL. After being lightly mixed and centrifuged at a low speed, the mixture was incubated at 25 °C for 5 min and at 42 °C for 25 min, and it was immediately heated to 85 °C for 5 s to stop the reaction. Finally, 180 µL of nuclease-free water was added to each 20 µL cDNA synthesis reaction, and this was stored at −20 °C until the analysis.

Real-time quantitative PCR was run to detect the mRNA expression levels of related genes in liver tissues. Quantitative analysis of PCR was carried out on the ABI StepOne Plus Real-Time PCR System (Applied Biosystems, Life Technologies, Gaithersburg, MD, USA) by using a Servicebio^®^ 2×Universal Blue SYBR Green qPCR Master Mix kit (Cat. No. G3326, Wuhan Servicebio Technology Co., LTD., Hubei, China). The primer sequences for the target genes are shown in Table 1. The cDNA generated from each sample was used as a template, and the reaction system was 20 μL, as follows: 2×qPCR Mix 10 μL, 10 μmol/L forward and reverse primers 0.4 μL, cDNA 2 μL, nuclease-free water 7.2 μL. The PCR reaction conditions were performed with the predenaturation phase of the template at 95 °C for 30 s, and a total of 40 cycles were used for the template amplification phase at 95 °C for 15 s and at 60 °C for 30 s; finally, the temperature was slowly increased by 0.3 °C every 10 s from 60 to 95 °C. All reactions were set to three replicates, and the relative fold difference in the mRNA expression levels was calculated by using the 2^−ΔΔCT^ method [32] with GAPDH as an internal reference.

### 2.6. Statistical Analysis

Statistical analysis was performed by using the SPSS software (version 22, Chicago, IL, USA). Significant differences between the LPS and CON groups were determined by using one-way analysis of variance, and probability values of ≤0.05 were taken to indicate significance. All data were presented as the means ± standard deviation, and Graph Prism 8.0 software (GraphPad, San Diego, CA, USA) was used to generate the corresponding graphs.

## 3. Results

### 3.1. Changes in Serum Biochemical Indexes

**Figure 1:**
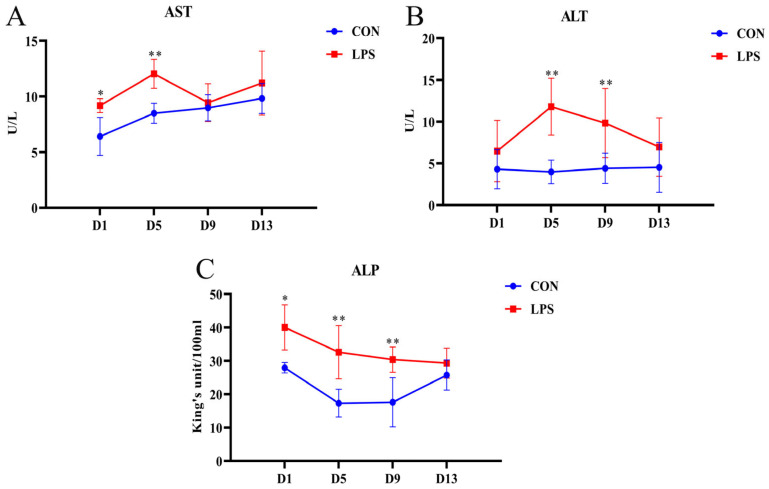
The dynamic changes in the serum enzyme activity of weaned piglets induced by LPS. Note: (**A**) AST, aspartate aminotransferase; (**B**) ALT, alanine aminotransferase; (**C**) ALP, alkaline phosphatase. “*” indicates a significant difference (*p* < 0.05), and “**” indicates an extremely significant difference (*p* < 0.01).

The serum AST, ALT, and ALP levels can be used as biochemical indicators of liver injury, and the data for the enzyme activity in the serum are presented in Figure 1. Compared with those in the CON group, the activities of AST, ALT, and ALP increased in the LPS group. On the first and fifth days, the AST activity in the LPS group significantly increased (Figure 1A, *p* = 0.025, *p* = 0.002). On the fifth and ninth days, the ALT level in the LPS group was higher than that in the CON group (Figure 1B, *p* = 0.001, *p* = 0.006), and the ALP activity in the LPS group also increased, especially on the first, fifth, and ninth days (Figure 1C, *p* = 0.011, *p* = 0.001, *p* = 0.002). However, on the thirteenth day after LPS induction, there were no significant differences in the activities of the three enzymes between the LPS group and the CON group (all *p* > 0.05).

### 3.2. Antioxidant Capacity

#### 3.2.1. Changes in Serum Antioxidant Parameters

The serum antioxidant parameters of the weaned piglets in the CON group and LPS group are shown in Table 2. The changes in serum antioxidant enzymes and MDA content reflect the whole body’s ability to resist oxidative stress. Compared with the CON group, the serum T-AOC activity decreased significantly on the first and fifth days after LPS induction (*p* = 0.006, *p* = 0.032), and the SOD and GSH-Px activities decreased significantly on the fifth day after LPS induction (SOD: *p* = 0.034; GSH-Px: *p* = 0.003). However, there were no significant differences in the CAT activity during the 13 days of continuous induction (all *p* > 0.05), and the MDA level in the LPS group was significantly higher than that in the CON group on the thirteenth day (*p* = 0.039).

#### 3.2.2. Changes in the Antioxidant Parameters of Livers

The antioxidant parameters of the liver are shown in Table 3. On the first and fifth days, the activities of SOD and CAT significantly increased in the liver homogenate of the LPS group (SOD: *p* = 0.017, *p* = 0.035; CAT: *p* = 0.009, *p* = 0.023). Compared with those in the CON group, the activities of T-AOC and GSH-Px in the LPS group significantly decreased on the fifth day (T-AOC: *p* = 0.047; GSH-Px: *p* = 0.025), while the content of MDA in the livers of the LPS group did not significantly change during the 13 days of LPS induction (all *p* > 0.05).

### 3.3. Histopathological Observation of Livers

**Figure 2:**
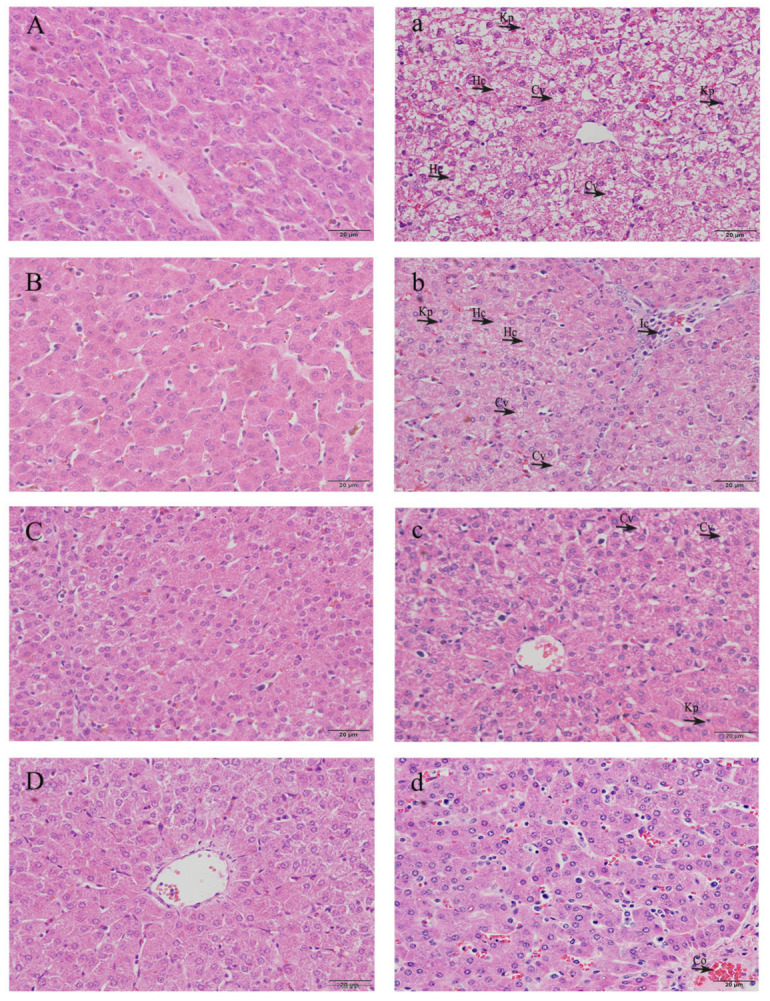
Effects of LPS induction on the liver microstructure of the weaned piglets. Note: Original magnification: 400×. Scale bars = 200 μm. (1) CON group: (**A**–**D**) show the liver of saline-treated weaned piglets on days 1, 5, 9, and 13, respectively; (2) LPS group: (**a**–**d**) show the liver of LPS-treated weaned piglets on days 1, 5, 9, and 13, respectively. Hc, hepatocyte caryolysis; Kp, karyopycnosis; Cv, cytoplasm vacuolization; Ic, inflammatory cell infiltration; Co, congestion.

In order to evaluate the dynamic histopathological changes in livers after LPS induction, the liver sections were stained with H&E and observed under a light microscope. According to the results (Figure 2), the morphology and structure of the hepatocytes of the liver tissues were normal in the CON group (Figure 2A–D), the outline of the liver lobule was clear, the hepatic cord was arranged radially, and the nucleus was located in the center of the cell. Compared with weaned piglets injected with saline at the same age, morphological changes related to liver injury were observed in piglets from the LPS group. On the first and fifth days after LPS induction (Figure 2a,b), the microstructures demonstrated hepatocyte caryolysis, hepatocyte vacuolization, karyopycnosis, disordered hepatic cell cord arrangement, some inflammatory cells that infiltrated around the central vein, and the appearance of congestion. On the ninth and thirteenth days (Figure 2c,d), the damage to hepatocytes in the LPS group was alleviated, with only a small amount of inflammatory cell infiltration and mild congestion.

### 3.4. Gene mRNA Expression in Livers

**Figure 3:**
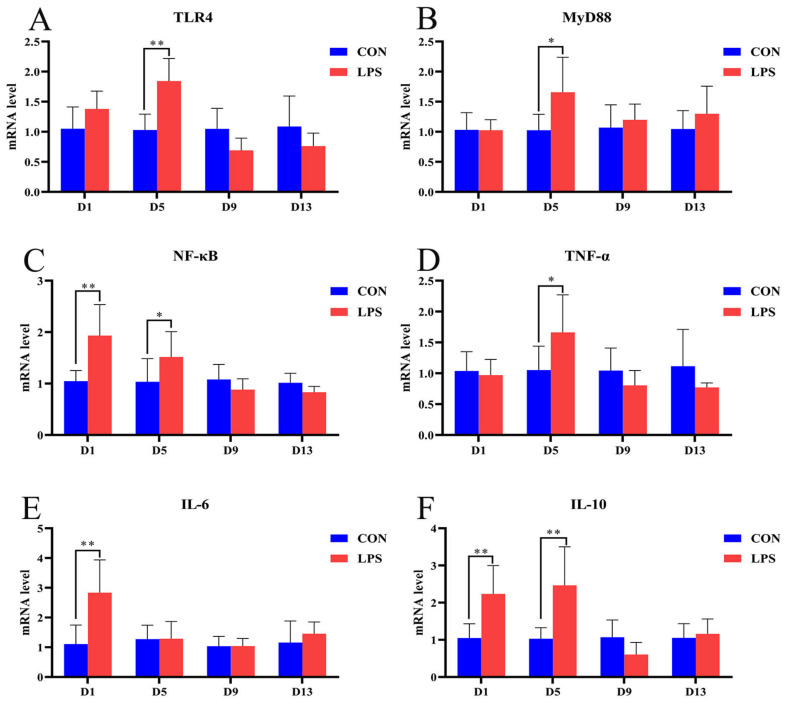
Effects of LPS induction on the TLR4/MyD88/NF-κB signal and inflammatory cytokines in livers. Note: (**A**) TLR4, toll-like receptor 4; (**B**) MyD88, myeloid differentiation factor 88; (**C**) NF-κB, nuclear factor-κB; (**D**) TNF-α, tumor necrosis factor-alpha; (E) IL-6, interleukin-6; (F) IL-10, interleukin-10. Data represent the mean ± standard deviation (n = 6 in each group), “*” indicates a significant difference (*p* < 0.05), and “**” indicates an extremely significant difference (*p* < 0.01).

To investigate the effects of LPS-induced liver injury and inflammatory cytokines in weaned piglets, the mRNA expression levels of TLR4, MyD88, NF-κB, TNF-α, IL-6, and IL-10 were detected in the livers with real-time PCR, as shown in Figure 3. Compared with the CON group, the mRNA expression of NF-κB, IL-6, and IL-10 in the weaned piglets of the LPS group was significantly upregulated on the first day (NF-κB: *p* = 0.001; IL-6: *p* = 0.001; IL-10: *p* = 0.007). On the fifth day, the mRNA expression of TLR4, MyD88, NF-κB, TNF-α, and IL-10 was significantly upregulated (TLR4: *p* = 0.003; MyD88: *p* = 0.018; NF-κB: *p* = 0.029; TNF-α: *p* = 0.02; IL-10: *p* = 0.001). However, there were no significant differences on the ninth and thirteenth days (all *p* > 0.05).

## 4. Discussion

The similarities between pigs and humans in terms of their anatomy, physiology, immunology, and genome enhance their potential as human models [33,34,35]. It is well known that blood biochemical indicators reflect cell permeability and metabolic function, which are key indicators of body health [36]. In the current study, we evaluated the dynamic effects of LPS induction on serum enzyme activities associated with liver damage in a weaned piglet model. AST, ALT, and ALP can be regarded as the main characteristic substances of liver injury, and they are mainly located in the cytoplasm and are released into the blood circulation after cell injury [37]. Therefore, an increase in ALT, AST, and ALP activities in the blood is usually a sign of stress response, and the serum levels of these intracellular enzymes are useful quantitative markers that indicate the degree and type of hepatocellular damage [38]. In this study, the activities of serum AST, ALT, and ALP in the LPS group were significantly higher than those in the CON group, which suggested that LPS induction caused liver damage in piglets. Our results are in line with those of Khan et al. [39], who reported the serum levels of ALT and AST in Sprague Dawley rats; compared with the control group, the levels of AST and ALT in the LPS-treated group were higher. Xu et al. [40] found that liver tissue was significantly damaged and serum ALT, AST, and ALP activities were significantly increased in LPS-treated mice. Similarly, Xu et al. [41] reported that an LPS challenge increased the serum AST activity and the AST/ALT ratio of weaned piglets within 24 h of LPS induction, and it reached the peak at 8 h.

Previous reports have shown that LPS exposure could result in oxidative stress by increasing ROS formation [42,43]. Oxidative stress refers to the overproduction of free radicals, such as ROSs and reactive nitrogen free radicals, which damage biofilm lipids, proteins, DNA, and other macromolecules [44]. Generally, the body can remove excessive free radicals through the enzymes of the antioxidant defense system to protect the body from oxidative damage [45], and oxidative damage can be judged by measuring the activity of antioxidant enzymes and the content of MDA. Among them, T-AOC, as a comprehensive index for evaluating the antioxidant system, reflects the cumulative effect of antioxidants in the body, and the higher the activity in a certain range is, the stronger the body’s antioxidant capacity is [46]. SOD, CAT, and GSH-Px are considered the first line of defense of the antioxidant enzyme system against ROSs produced in the process of oxidative stress [47]. MDA is a lipid peroxide produced by the reaction of ROSs and polyunsaturated fatty acids in vivo. Its content can reflect the degree of oxidative damage, and excessive production will cause cytotoxicity, so it is also one of the main biochemical indicators for measuring oxidative stress in animals [48,49].

In the present study, LPS induction was able to significantly reduce the activities of T-AOC and GSH-Px in the serum and liver, and it significantly increased the content of MDA in the serum. Interestingly, the SOD and CAT activities in the livers of the LPS group were higher than those in the livers of the CON group, while the SOD activity in the serum was lower than that of the CON group. The above results were most obvious on the first and fifth days of LPS induction. Li et al. [29] reported that an LPS challenge increased the level of MDA and the activities of CAT and SOD in the livers of weaned piglets, which was almost the same as our results. The increase in the CAT and SOD activities in livers may be attributed to the preventive measures taken by the host to cope with the superoxide anion load after an LPS challenge and the need for the body to express more antioxidant enzymes in the liver to eliminate the increased oxidant burden [50]. However, due to the challenge of exogenous LPS and the changes in antioxidant enzymes in the body, this suggests that oxidative stress was induced in the piglets by LPS. Similarly, Li et al. [15] showed that LPS administration significantly changed the plasma MDA, CAT, GSH-Px, and T-AOC levels in piglets at 4 h post-challenge, which proved that an animal model of LPS-induced oxidative stress was successfully established, and that the oxidative stress induced by LPS could be resisted with a dietary exogenous catalase supplement.

Some studies have shown that TLR4 is expressed in various liver tissues and cells, and the TLR4-mediated MyD88 signaling pathway can regulate the innate immunity of the body and participate in the pathological response of liver tissue [51]. As a pattern recognition receptor for innate immunity, TLR4 can specifically recognize LPS from the outer membrane of Gram-negative bacteria [52]. First of all, LPS-binding protein (LBP) can recognize LPS and form a ternary complex of LPS¬–LBP–CD14 with membrane CD14 on the surface of myeloid-derived cells; then, it can be transported to the protein complex of TLR4–MD2, and the ternary complex combines with TLR4 with the help of MD-2 to activate TLR4 [51,53]. With the activation of the TLR4 signal, NF-κB is constantly activated, and the expression of related genes in the nucleus is abnormally increased, producing inflammatory factors such as IL-1β, IL-6, and TNF-α, which cause chemotaxis and aggregation of inflammatory cells [54]; then, the released cytokines can , in turn, act again and activate NF-κB, thus forming a positive feedback regulation and amplifying the inflammatory reaction cascade [55,56]. TNF-α, IL-6, and IL-10 are important inflammatory cytokines that play related roles in inflammatory diseases and are involved in LPS-induced liver injury [57,58,59]. Zhang et al. [30] found that LPS-induced upregulation of the expression levels of proinflammatory cytokines including TNF-α, IL-1β, and IL-6 in the livers of weaned piglets and the supplementation of fish oil in feed could alleviate LPS-induced liver inflammation. Therefore, in order to further explore the molecular mechanism of LPS-induced liver inflammation response, the mRNA expression related to the TLR4 signaling pathway and inflammatory cytokines was examined here. The mRNA expression of TLR4, MyD88, NF-κB, TNF-α, IL-6, and IL-10 was upregulated, especially on the first and fifth days of LPS induction, which was consistent with the histopathological changes in liver morphology. However, there were no significant differences between the LPS group and CON group on the ninth and thirteenth days. This indicates that LPS-induced liver inflammation may be regulated by the TLR4 pathway, and continuous LPS induction may trigger the body’s endotoxin tolerance, which can prevent the fatal challenge of LPS [60,61].

## 5. Conclusions

At the early stage of LPS induction, the body experiences oxidative stress and mild liver injury, and the liver damage gradually weakens at later stages of LPS induction, which may be attributed to endotoxin tolerance and hepatic immunomodulatory function. Obviously, this provides a kind of novel evidence for the liver’s pathological mechanism in weaned piglets that are challenged by a Gram-negative bacterial infection. In the future, further studies are needed to clearly understand the interactions of LPS, inflammation, and oxidative stress in liver disease.

## Figures and Tables

**Table 1:** Primers used in the real-time PCR analysis.

Gene	Primer Sequence (5′→3′)	Product Size (bp)	GenBank No.
GAPDH	F: CCTGGAGAAACCTGCAAAATA R: AACCTGGTCCTCAGTGTAGCC	100	NM_001206359.1
TLR4	F: GACGAAGACTGGGTGAGGAATGAAC R: CCTGGATGATGTTAGCAGCGATGG	124	NM_001113039.2
MyD88	F: CGTCTGGTCCATTGCTAGAACTC R: TTCTGATGGGCACCTGGAGAGAG	141	NM_001099923.1
NF-κB	F: CTGAGGCTATAACTCGCTTGGTGAC R: CATGTCCGCAATGGAGGAGAAGTC	131	NM_001114281.1
IL-6	F: ATAAGGGAAATGTCGAGGCTGTGC R: GGGTGGTGGCTTTGTCTGGATTC	93	NM_001252429.1
TNF-α	F: TCTATTTTGGGATCATTGCCC R: CCAGCCCCTCATTCTCTTTCT	127	NM_214022.1
IL-10	F: GCATCCACTTCCCAACCA R: GCAACAAGTCGCCCATCT	108	NM_214041.1

F: Forward primer; R: Reverse primer.

**Table 2:** Effects of LPS induction on the serum antioxidant parameters of weaned piglets.

Items	Group	D1	D5	D9	D13
T-AOC (mM)	CON	0.22 ± 0.020	0.21 ± 0.008	0.19 ± 0.019	0.19 ± 0.039
	LPS	0.16 ± 0.020 ##	0.16 ± 0.025 #	0.21 ± 0.037	0.19 ± 0.035
SOD (U/mL)	CON	28.01 ± 2.48	27.73 ± 1.19	27.40 ± 0.89	29.29 ± 0.67
	LPS	27.65 ± 2.56	24.73 ± 1.59 #	29.92 ± 1.24	31.86 ± 0.79
GSH-Px (U/mL)	CON	270.80 ± 30.46	317.20 ± 28.96	260.60 ± 34.96	241.48 ± 22.81
	LPS	295.86 ± 34.13	245.63 ± 21.37 ##	258.68 ± 15.93	216.05 ± 28.98
CAT (U/mL)	CON	6.52 ± 0.98	6.86 ± 1.88	6.24 ± 2.24	7.19 ± 2.21
	LPS	8.58 ± 0.49	6.91 ± 1.33	6.73 ± 1.86	5.97 ± 0.75
MDA (nmol/mL)	CON	2.60 ± 0.24	2.77 ± 0.72	2.65 ± 0.17	2.49 ± 0.26
	LPS	2.31 ± 0.22	2.38 ± 0.30	3.16 ± 0.74	3.21 ± 0.83 *

Note: T-AOC, total antioxidant capacity; SOD, superoxide dismutase; CAT, catalase; GSH-Px, glutathione peroxidase; MDA, malondialdehyde. “*” or “#” indicates a significant increase or decrease (*p* < 0.05), and “##” indicates an extremely significant increase or decrease (*p* < 0.01).

**Table 3:** Effects of LPS induction on the antioxidant parameters in the livers of weaned piglets.

Items	Group	D1	D5	D9	D13
T-AOC (mmol/gprot)	CON	0.110 ± 0.021	0.093 ± 0.018	0.061 ± 0.004	0.093 ± 0.035
	LPS	0.086 ± 0.003	0.065 ± 0.008 #	0.063 ± 0.005	0.075 ± 0.012
SOD (U/mgprot)	CON	95.43 ± 14.18	101.21 ± 11.72	102.35 ± 13.52	102.52 ± 15.03
	LPS	116.62 ± 2.74 *	119.57 ± 4.03 *	97.40 ± 7.63	95.88 ± 7.12
GSH-Px (U/mgprot)	CON	39.67 ± 7.86	45.24 ± 7.11	44.47 ± 4.17	33.29 ± 6.20
	LPS	41.41 ± 3.71	33.63 ± 0.44 #	51.24 ± 3.68	32.76 ± 8.74
CAT (U/mgprot)	CON	26.21 ± 5.06	26.88 ± 7.38	33.84 ± 1.25	28.34 ± 12.40
	LPS	39.54 ± 5.89 **	38.20 ± 6.99 *	36.53 ± 3.32	20.14 ± 2.00
MDA (nmol/mgprot)	CON	0.86 ± 0.09	0.48 ± 0.24	0.60 ± 0.09	0.72 ± 0.16
	LPS	0.62 ± 0.15	0.55 ± 0.07	0.55 ± 0.17	0.65 ± 0.05

Note: T-AOC, total antioxidant capacity; SOD, superoxide dismutase; CAT, catalase; GSH-Px, glutathione peroxidase; MDA, malondialdehyde. “*” or “#” indicates a significant increase or decrease (*p* < 0.05), and “**” indicates an extremely significant increase or decrease (*p* < 0.01).

## Data Availability

All data appear in the manuscript. For further inquiries, please contact the first author or corresponding author.

## References

[1] Dhainaut J.F., Marin N., Mignon A., Vinsonneau C. (2001). Hepatic response to sepsis: Interaction between coagulation and inflammatory processes. Crit. Care Med..

[2] Zhang Y., Xue W., Zhang W., Yuan Y., Zhu X., Wang Q., Wei Y., Yang D., Yang C., Chen Y. (2020). Histone methyltransferase G9a protects against acute liver injury through GSTP1. Cell Death Differ..

[3] Compare D., Coccoli P., Rocco A., Nardone O.M., De Maria S., Carteni M., Nardone G. (2012). Gut-liver axis: The impact of gut microbiota on non alcoholic fatty liver disease. Nutr. Metab. Cardiovasc. Dis..

[4] Kubes P., Jenne C. (2018). Immune Responses in the Liver. Annu. Rev. Immunol..

[5] Hasuda A.L., Person E., Khoshal A.K., Bruel S., Puel S., Oswald I.P., Bracarense A., Pinton P. (2022). Deoxynivalenol induces apoptosis and inflammation in the liver: Analysis using precision-cut liver slices. Food Chem. Toxicol..

[6] Seo H.Y., Kim M.K., Lee S.H., Hwang J.S., Park K.G., Jang B.K. (2018). Kahweol Ameliorates the Liver Inflammation through the Inhibition of NF-κB and STAT3 Activation in Primary Kupffer Cells and Primary Hepatocytes. Nutrients.

[7] Seki E., Schnabl B. (2012). Role of innate immunity and the microbiota in liver fibrosis: Crosstalk between the liver and gut. J. Physiol..

[8] Hamesch K., Borkham-Kamphorst E., Strnad P., Weiskirchen R. (2015). Lipopolysaccharide-induced inflammatory liver injury in mice. Lab. Anim..

[9] Strnad P., Tacke F., Koch A., Trautwein C. (2017). Liver-guardian, modifier and target of sepsis. Nat. Rev. Gastroenterol. Hepatol..

[10] Kaur G., Tirkey N., Chopra K. (2006). Beneficial effect of hesperidin on lipopolysaccharide-induced hepatotoxicity. Toxicology.

[11] Elazab M.F.A., Nasr N.E., Ahmed M.S., Alrashdi B.M., Dahran N., Alblihed M.A., Elmahallawy E.K. (2022). The Effects of Bacterial Lipopolysaccharide (LPS) on Turkey Poults: Assessment of Biochemical Parameters and Histopathological Changes. Vet. Sci..

[12] Czaja A.J. (2014). Hepatic inflammation and progressive liver fibrosis in chronic liver disease. World J. Gastroenterol..

[13] Jaeschke H. (2000). Reactive oxygen and mechanisms of inflammatory liver injury. J. Gastroenterol. Hepatol..

[14] Mittal M., Siddiqui M.R., Tran K., Reddy S.P., Malik A.B. (2014). Reactive oxygen species in inflammation and tissue injury. Antioxid. Redox Signal.

[15] Li Y., Zhao X., Jiang X., Chen L., Hong L., Zhuo Y., Lin Y., Fang Z., Che L., Feng B. (2020). Effects of dietary supplementation with exogenous catalase on growth performance, oxidative stress, and hepatic apoptosis in weaned piglets challenged with lipopolysaccharide. J. Anim. Sci..

[16] Hall D.M., Buettner G.R., Oberley L.W., Xu L., Matthes R.D., Gisolfi C.V. (2001). Mechanisms of circulatory and intestinal barrier dysfunction during whole body hyperthermia. Am. J. Physiol. Heart. Circ. Physiol..

[17] Muccioli G.G., Naslain D., Backhed F., Reigstad C.S., Lambert D.M., Delzenne N.M., Cani P.D. (2010). The endocannabinoid system links gut microbiota to adipogenesis. Mol. Syst. Biol..

[18] Robinson M.W., Harmon C., O’Farrelly C. (2016). Liver immunology and its role in inflammation and homeostasis. Cell Mol. Immunol..

[19] Newton K., Dixit V.M. (2012). Signaling in innate immunity and inflammation. Cold Spring Harb. Perspect. Biol..

[20] Feng G., Zheng K., Cao T., Zhang J., Lian M., Huang D., Wei C., Gu Z., Feng X. (2018). Repeated stimulation by LPS promotes the senescence of DPSCs via TLR4/MyD88-NF-κB-p53/p21 signaling. Cytotechnology.

[21] Sabroe I., Parker L.C., Dower S.K., Whyte M.K. (2008). The role of TLR activation in inflammation. J. Pathol..

[22] Tanimura N., Saitoh S., Matsumoto F., Akashi-Takamura S., Miyake K. (2008). Roles for LPS-dependent interaction and relocation of TLR4 and TRAM in TRIF-signaling. Biochem. Biophys. Res. Commun..

[23] Motshwene P.G., Moncrieffe M.C., Grossmann J.G., Kao C., Ayaluru M., Sandercock A.M., Robinson C.V., Latz E., Gay N.J. (2009). An oligomeric signaling platform formed by the Toll-like receptor signal transducers MyD88 and IRAK-4. J. Biol. Chem..

[24] Kim I.D., Ha B.J. (2010). The effects of paeoniflorin on LPS-induced liver inflammatory reactions. Arch. Pharm. Res..

[25] Larrosa M., Azorin-Ortuno M., Yanez-Gascon M.J., Garcia-Conesa M.T., Tomas-Barberan F., Espin J.C. (2011). Lack of effect of oral administration of resveratrol in LPS-induced systemic inflammation. Eur. J. Nutr..

[26] Carroll J.A., Touchette K.J., Matteri R.L., Dyer C.J., Allee G.L. (2002). Effect of spray-dried plasma and lipopolysaccharide exposure on weaned pigs: II. Effects on the hypothalamic-pituitary-adrenal axis of weaned pigs. J. Anim. Sci..

[27] Zhu C., Wu Y., Jiang Z., Zheng C., Wang L., Yang X., Ma X., Gao K., Hu Y. (2015). Dietary soy isoflavone attenuated growth performance and intestinal barrier functions in weaned piglets challenged with lipopolysaccharide. Int. Immunopharmacol..

[28] Tsai T.H., Tam K., Chen S.F., Liou J.Y., Tsai Y.C., Lee Y.M., Huang T.Y., Shyue S.K. (2018). Deletion of caveolin-1 attenuates LPS/GalN-induced acute liver injury in mice. J. Cell Mol. Med..

[29] Li Q., Liu Y., Che Z., Zhu H., Meng G., Hou Y., Ding B., Yin Y., Chen F. (2012). Dietary L-arginine supplementation alleviates liver injury caused by *Escherichia coli* LPS in weaned pigs. Innate Immun..

[30] Zhang J., Xu X., Zhu H., Wang Y., Hou Y., Liu Y. (2019). Dietary fish oil supplementation alters liver gene expressions to protect against LPS-induced liver injury in weanling piglets. Innate Immun..

[31] Feldman A.T., Wolfe D. (2014). Tissue processing and hematoxylin and eosin staining. Methods Mol. Biol..

[32] Livak K.J., Schmittgen T.D. (2001). Analysis of relative gene expression data using real-time quantitative PCR and the 2(-Delta Delta C(T)) Method. Methods.

[33] Nykonenko A., Vávra P., Zonča P. (2017). Anatomic Peculiarities of Pig and Human Liver. Exp. Clin. Transplant..

[34] Simon G.A., Maibach H.I. (2000). The pig as an experimental animal model of percutaneous permeation in man: Qualitative and quantitative observations--an overview. Skin Pharmacol. Appl. Skin Physiol..

[35] Lunney J.K., Van Goor A., Walker K.E., Hailstock T., Franklin J., Dai C. (2021). Importance of the pig as a human biomedical model. Sci. Transl. Med..

[36] Kwo P.Y., Cohen S.M., Lim J.K. (2017). ACG Clinical Guideline: Evaluation of Abnormal Liver Chemistries. Am. J. Gastroenterol..

[37] Nyblom H., Berggren U., Balldin J., Olsson R. (2004). High AST/ALT ratio may indicate advanced alcoholic liver disease rather than heavy drinking. Alcohol Alcohol..

[38] Mitra S.K., Venkataranganna M.V., Sundaram R., Gopumadhavan S. (1998). Protective effect of HD-03, a herbal formulation, against various hepatotoxic agents in rats. J. Ethnopharmacol..

[39] Khan H.U., Aamir K., Jusuf P.R., Sethi G., Sisinthy S.P., Ghildyal R., Arya A. (2021). Lauric acid ameliorates lipopolysaccharide (LPS)-induced liver inflammation by mediating TLR4/MyD88 pathway in Sprague Dawley (SD) rats. Life Sci..

[40] Xu Q., Xu J., Zhang K., Zhong M., Cao H., Wei R., Jin L., Gao Y. (2021). Study on the protective effect and mechanism of Dicliptera chinensis (L.) Juss (Acanthaceae) polysaccharide on immune liver injury induced by LPS. Biomed. Pharmacother..

[41] Xu Q., Guo J., Li X., Wang Y., Wang D., Xiao K., Zhu H., Wang X., Hu C.A., Zhang G. (2021). Necroptosis Underlies Hepatic Damage in a Piglet Model of Lipopolysaccharide-Induced Sepsis. Front. Immunol..

[42] Yang H., Lv H., Li H., Ci X., Peng L. (2019). Oridonin protects LPS-induced acute lung injury by modulating Nrf2-mediated oxidative stress and Nrf2-independent NLRP3 and NF-κB pathways. Cell Commun. Signal..

[43] Wu H., Wang Y., Zhang Y., Xu F., Chen J., Duan L., Zhang T., Wang J., Zhang F. (2020). Breaking the vicious loop between inflammation, oxidative stress and coagulation, a novel anti-thrombus insight of nattokinase by inhibiting LPS-induced inflammation and oxidative stress. Redox Biol..

[44] Neubauer O., Reichhold S., Nics L., Hoelzl C., Valentini J., Stadlmayr B., Knasmüller S., Wagner K.-H. (2010). Antioxidant responses to an acute ultra-endurance exercise: Impact on DNA stability and indications for an increased need for nutritive antioxidants in the early recovery phase. Br. J. Nutr..

[45] Zhao D., Wu T., Yi D., Wang L., Li P., Zhang J., Hou Y., Wu G. (2017). Dietary Supplementation with Lactobacillus casei Alleviates Lipopolysaccharide-Induced Liver Injury in a Porcine Model. Int. J. Mol. Sci..

[46] Saita E., Kondo K., Momiyama Y. (2015). Anti-Inflammatory Diet for Atherosclerosis and Coronary Artery Disease: Antioxidant Foods. Clin. Med. Insights Cardiol..

[47] Cecerska-Heryć E., Surowska O., Heryć R., Serwin N., Napiontek-Balińska S., Dołęgowska B. (2021). Are antioxidant enzymes essential markers in the diagnosis and monitoring of cancer patients—A review. Clin. Biochem..

[48] Celi P. (2011). Biomarkers of oxidative stress in ruminant medicine. Immunopharm. Immunot..

[49] Romero F.J., Bosch-Morell F., Romero M.J., Jareño E.J., Romero B., Marín N., Romá J. (1998). Lipid peroxidation products and antioxidants in human disease. Environ. Health Perspect..

[50] Nandi D., Mishra M.K., Basu A., Bishayi B. (2010). Protective effects of interleukin-6 in lipopolysaccharide (LPS)-induced experimental endotoxemia are linked to alteration in hepatic anti-oxidant enzymes and endogenous cytokines. Immunobiology.

[51] Akira S., Takeda K., Kaisho T. (2001). Toll-like receptors: Critical proteins linking innate and acquired immunity. Nat. Immunol..

[52] Dunzendorfer S., Lee H.K., Soldau K., Tobias P.S. (2004). TLR4 is the signaling but not the lipopolysaccharide uptake receptor. J. Immunol..

[53] Jerala R. (2007). Structural biology of the LPS recognition. Int. J. Med. Microbiol..

[54] Doyle S.L., O'Neill L.A. (2006). Toll-like receptors: From the discovery of NFκB to new insights into transcriptional regulations in innate immunity. Biochem. Pharmacol..

[55] Li Q., Verma I.M. (2002). NF-κB regulation in the immune system. Nat. Rev. Immunol..

[56] Peng J., He Q., Li S., Liu T., Zhang J. (2022). Hydrogen-Rich Water Mitigates LPS-Induced Chronic Intestinal Inflammatory Response in Rats via Nrf-2 and NF-κB Signaling Pathways. Vet. Sci..

[57] Zhong W., Qian K., Xiong J., Ma K., Wang A., Zou Y. (2016). Curcumin alleviates lipopolysaccharide induced sepsis and liver failure by suppression of oxidative stress-related inflammation via PI3K/AKT and NF-κB related signaling. Biomed. Pharmacother..

[58] Bradley J.R. (2008). TNF-mediated inflammatory disease. J. Pathol..

[59] Mietto B.S., Kroner A., Girolami E.I., Santos-Nogueira E., Zhang J., David S. (2015). Role of IL-10 in Resolution of Inflammation and Functional Recovery after Peripheral Nerve Injury. J. Neurosci..

[60] Yan J., Bai J., Gao C., Liang Y., Zhao B., Bian Y. (2017). Chronic unpredictable stress abrogates the endotoxin tolerance induced by repeated peripheral LPS challenge via the TLR4 signaling pathway. Neurosci. Lett..

[61] Cavaillon J.M., Adrie C., Fitting C., Adib-Conquy M. (2003). Endotoxin tolerance: Is there a clinical relevance?. J. Endotoxin Res..

